# Novel Rare Missense Variations and Risk of Autism Spectrum Disorder: Whole-Exome Sequencing in Two Families with Affected Siblings and a Two-Stage Follow-Up Study in a Japanese Population

**DOI:** 10.1371/journal.pone.0119413

**Published:** 2015-03-25

**Authors:** Jun Egawa, Yuichiro Watanabe, Chenyao Wang, Emiko Inoue, Atsunori Sugimoto, Toshiro Sugiyama, Hirofumi Igeta, Ayako Nunokawa, Masako Shibuya, Itaru Kushima, Naoki Orime, Taketsugu Hayashi, Takashi Okada, Yota Uno, Norio Ozaki, Toshiyuki Someya

**Affiliations:** 1 Department of Psychiatry, Niigata University Graduate School of Medical and Dental Sciences, Niigata, Japan; 2 Department of Pediatric Psychiatry, Center for Transdisciplinary Research, Niigata University, Niigata, Japan; 3 Division of Medical Education, Comprehensive Medical Education Center, School of Medicine, Faculty of Medicine, Niigata University, Niigata, Japan; 4 Department of Psychiatry, Nagoya University Graduate School of Medicine, Nagoya, Aichi, Japan; 5 Department of Child and Adolescent Psychiatry, Hamamatsu University School of Medicine, Hamamatsu, Shizuoka, Japan; 6 Oojima Hospital, Sanjo, Niigata, Japan; 7 Health Administration Center, Headquarters for Health Administration, Niigata University, Niigata, Japan; Rikagaku Kenkyūsho Brain Science Institute, JAPAN

## Abstract

Rare inherited variations in multiplex families with autism spectrum disorder (ASD) are suggested to play a major role in the genetic etiology of ASD. To further investigate the role of rare inherited variations, we performed whole-exome sequencing (WES) in two families, each with three affected siblings. We also performed a two-stage follow-up case-control study in a Japanese population. WES of the six affected siblings identified six novel rare missense variations. Among these variations, *CLN8* R24H was inherited in one family by three affected siblings from an affected father and thus co-segregated with ASD. In the first stage of the follow-up study, we genotyped the six novel rare missense variations identified by WES in 241 patients and 667 controls (the Niigata sample). Only *CLN8* R24H had higher mutant allele frequencies in patients (1/482) compared with controls (1/1334). In the second stage, this variation was further genotyped, yet was not detected in a sample of 309 patients and 350 controls (the Nagoya sample). In the combined Niigata and Nagoya samples, there was no significant association (odds ratio = 1.8, 95% confidence interval = 0.1–29.6). These results suggest that *CLN8* R24H plays a role in the genetic etiology of ASD, at least in a subset of ASD patients.

## Introduction

Autism spectrum disorder (ASD) is a neurodevelopmental disorder marked by social and communication deficits and the presence of rigid and repetitive behaviors and interests.

ASD is a complex disorder with an estimated heritability of 52.4%, mostly due to common variations, although rare variations also contribute substantially to ASD liability [1]. A whole-exome sequencing (WES) study of 2517 simplex families shows that rare *de novo* mutations contribute to the genetic etiology of ASD [2], while WES studies of multiplex families suggest that rare inherited variations also play an important role in the genetic etiology of ASD [3, 4]. In three consanguineous multiplex families, inherited homozygous missense variations co-segregate with ASD [3]. In 10 multiplex ASD families, the number of heterozygous truncating variations transmitted to affected siblings was significantly higher than those that were not transmitted [4].

To further investigate the role of rare inherited variations in the genetic etiology of ASD, we performed WES in two families, each with three affected siblings. We also performed a two-stage follow-up study in a Japanese population.

## Materials and Methods

### Ethics Statement

This study was approved by the Ethics Committee on Genetics of Niigata University School of Medicine, and the Ethics Committee of the Nagoya University Graduate School of Medicine and associated institutes and hospitals. Written informed consent was obtained from all participants and/or their families.

### Participants

**Fig 1:**
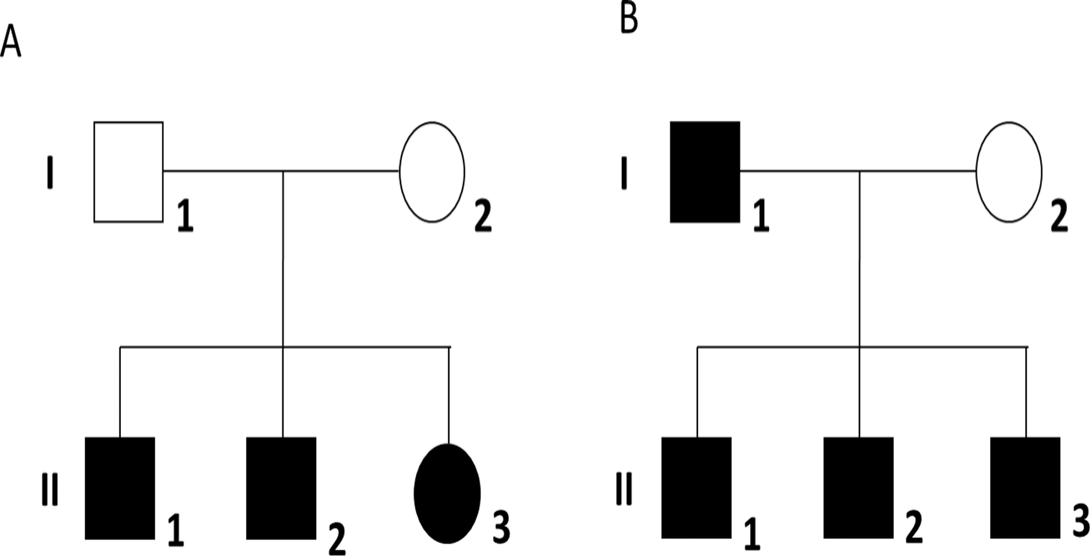
Pedigrees of two families, each with three autism spectrum disorder siblings. (A) Family #1. All three siblings (II-1, II-2, and II-3) were diagnosed with Asperger’s disorder. (B) Family #2. There were four affected individuals: a proband (II-1) with Asperger’s disorder, his brother (II-2) with Asperger’s disorder, his brother (II-3) with Asperger’s disorder and borderline intellectual functioning, and their father (I-1) with pervasive developmental disorder not otherwise specified. Shaded and unshaded symbols indicate affected and unaffected individuals, respectively. Squares and circles represent males and females, respectively.

All participants were of Japanese descent. We included two families, each with three ASD siblings, in a WES study. In family #1 (Fig. 1A), all three siblings (II-1, II-2, and II-3) were diagnosed with Asperger’s disorder. Their parents (I-1 and I-2) had not been diagnosed with a psychiatric disorder. In family #2 (Fig. 1B), there were four affected individuals: a proband (II-1) with Asperger’s disorder, his brother (II-2) with Asperger’s disorder, his brother (II-3) with Asperger’s disorder and borderline intellectual functioning, and their father (I-1) with pervasive developmental disorder not otherwise specified (PDD-NOS). The proband’s mother (I-2) has not been diagnosed with a psychiatric disorder. Participants were diagnosed according to Diagnostic and Statistical Manual of Mental Disorders, 4th Edition (DSM-IV) criteria. Diagnoses were made by experienced child psychiatrists based on all available information, including unstructured interviews of patients and their families, clinical observation and examination of medical records. We did not use standardized tests such as the Autism Diagnostic Interview-Revised (ADI-R) or the Autism Diagnostic Observation Schedule (ADOS).

Two independent case-control samples were used for the follow-up study (S1 Table). The Niigata sample in the first stage consisted of 241 patients with ASD (189 males and 52 females; mean age, 18.1 [SD 8.5] years) and 667 control individuals (341 males and 326 females; mean age, 38.3 [SD 10.8] years). Patient and control groups were not sex- or age-matched. Each participant was subjected to psychiatric assessment, as previously described [5, 6]. In brief, patients were diagnosed according to DSM-IV criteria for autistic disorder (*n* = 72), Asperger’s disorder (*n* = 105), or PDD-NOS (*n* = 64). Diagnoses were made by experienced child psychiatrists without ADI-R or ADOS. Controls were mainly recruited from hospital staff, and showed good social and occupational skills with no self-reported personal or family history (within first-degree relatives) of psychiatric disorders. However, these control subjects were not assessed using structured psychiatric interviews.

The Nagoya sample in the second stage consisted of 312 patients with ASD (236 males and 76 females; mean age, 19.6 [SD 10.2] years) and 352 control individuals (109 males and 243 females; mean age, 45.9 [SD 10.8] years). Patient and control groups were not sex- or age-matched. Cases were included if they met DSM-IV-TR criteria for autistic disorder (*n* = 131), Asperger’s disorder (*n* = 89), or PDD-NOS (*n* = 92). Control subjects were selected from the general population and had no history of mental disorders based on questionnaire responses from the subjects during the sample inclusion step.

### WES study

WES was performed on six affected individuals from family #1 (II-1, II-2, and II-3; Fig. 1A) and family #2 (II-1, II-2, and II-3; Fig. 1B) by the Contract Development & Manufacturing Center of Takara Bio Inc. (Mie, Japan). Exome libraries were prepared using the SureSelect Human All Exon V5 Kit (Agilent, Santa Clara, CA, USA) and sequenced using the HiSeq2000 system (Illumina, San Diego, CA, USA). Sequencing was performed in one lane of the HiSeq2000 using the paired-end module for 100-bp reads. Adaptor sequences were removed using cutadapt v1.2.1 (http://code.google.com/p/cutadapt/). Low quality reads were trimmed (quality threshold of 10 and minimum length of 32) and filtered out (minimum quality score of 10 and minimum percent of bases that must have quality of 99%) using FASTX-Toolkit v0.0.13 (http://hannonlab.cshl.edu/fastx_toolkit/). Paired-end sequence reads were retrieved using cmpfastq_pe (http://compbio.brc.iop.kcl.ac.uk/software/cmpfastq_pe.php) and mapped against the reference human genome (UCSC hg19) using the Burrows–Wheeler Aligner v0.5.9 (http://bio-bwa.sourceforge.net/). Polymerase chain reaction duplicates were removed using Picard v1.93 (http://picard.sourceforge.net/). Variation calling was performed using the Genome Analysis Toolkit v1.6–9 (http://www.broadinstitute.org/gatk/). Parameters of VariantFiltration for single nucleotide variations were set as follows: QD < 2.0, MQ < 40.0, FS > 60.0, HaplotypeScore > 13.0, MQRankSum < −12.5, and ReadPosRankSum < −8.0. Those for insertions/deletions were set as follows: QD < 2.0, FS > 200.0, InbreedingCoeff < −0.8, and ReadPosRankSum < −20.0. Variations were annotated using SnpEff v3.3h (http://snpeff.sourceforge.net/).

To prioritize variations, we applied several filtering steps. First, we filtered out variations with less than 10× coverage. Second, we included variations shared by the three affected siblings in each family. Third, we included putatively functional variations, including nonsense, frameshift and missense variations. Fourth, we included variations with Genomic Evolutionary Rate Profiling (GERP; http://mendel.stanford.edu/SidowLab/downloads/gerp/) scores > 5. Fifth, we filtered out variations registered in dbSNP Human BUID 138 (http://www.ncbi.nlm.nih.gov/projects/SNP/) or in the Human Genetic Variation Database 1.41 (http://www.genome.med.kyoto-u.ac.jp/SnpDB/).

To validate prioritized variations, Sanger sequencing was performed, as previously described [7]. Primer sequences for amplification are listed in S2 Table. Detailed information on amplification conditions is available upon request.

### Follow-up study

To determine if variations identified in the two families by WES contribute to genetic etiology of ASD, we performed a two-stage follow-up study. In the first stage, we genotyped six novel rare missense variations. In the second stage, we further genotyped one variation, identified in the first stage to have a higher mutant allele frequency in patients compared with controls. All variations were genotyped using the TaqMan 5′-exonuclease assay (Applied Biosystems, Foster City, CA, USA; S3 Table), as previously described [6]. To check the accuracy of the TaqMan assay, we genotyped six novel rare variations in the probands from families #1 and #2 using the TaqMan assay. In addition, Sanger sequencing was performed in heterozygous individuals for *CLN8* R24H determined by the TaqMan assay in the follow-up study.

Allelic associations were tested using Fisher’s exact test. Power calculations were performed using the Genetic Power Calculator (http://pngu.mgh.harvard.edu/~purcell/gpc/). Power was estimated using α = 0.05, assuming a disease prevalence of 0.01.

## Results

Almost all target regions (50.3 of 50.4 Mb) were captured in each exome (Table 1). Average read depth varied from 47.1× to 59.3×, and 95.4–97.2% of target regions were covered by 10 or more reads. A total of 185,015 and 178,706 sequence variations were called by WES in family #1 and family #2, respectively (Table 2). After several filtering steps, we prioritized four and two novel rare missense variations in family #1 and family #2, respectively. These variations were all confirmed by Sanger sequencing (Table 3). In family #1, the mutant alleles of all four variations (*SLC7A11* G77S, *ICA1* G167A, *DNAJC1* A508G, and *C1S* P19) were shown to be transmitted from the unaffected mother. In family #2, the *CLN8* R24H and *TRAPPC12* E297Q mutant alleles were transmitted from the affected father and unaffected mother, respectively.

**Table 1:** WES quality report summary.

	Family #1			Family #2		
	II-1	II-2	II-3	II-1	II-2	II-3
Captured target regions (Mb)	50.3	50.3	50.3	50.3	50.3	50.3
Average read depth	49.5	52.2	55.8	47.1	50.0	59.3
Percentage of target regions with coverage > 10×	95.9	96.4	97.2	95.4	96.2	97.2

WES, whole-exome sequencing.

**Table 2:** Filtering steps applied to variations called from WES of two families, each with three ASD siblings.

Filtering step	Number of remaining variations	
	Family #1	Family #2
Called	185,015	178,706
Covered by 10 or more reads	85,024	82,822
Shared by affected siblings	32,888	33,713
Putatively functional	3,374	3,508
Nonsense	13	12
Frameshift	40	44
Missense	3,321	3,452
GERP > 5	448	417
Novel	4	2

WES, whole-exome sequencing; ASD, autism spectrum disorder; GERP, Genomic Evolutionary Rate Profiling.

**Table 3:** Six novel rare missense variations identified by WES in two families, each with three ASD siblings.

Family	Chromosome	Position^a^	Allele^b^	Gene	Protein	GERP	Transmission
#1	4	139,162,995	C/T	*SLC7A11*	G77S	6.17	Mother
#1	7	8,258,011	C/G	*ICA1*	G167A	6	Mother
#1	10	22,048,172	G/C	*DNAJC1*	A508G	5.94	Mother
#1	12	7,172,475	C/T	*C1S*	P197S	6.17	Mother
#2	2	3,482,699	G/A	*TRAPPC12*	E297Q	5.05	Mother
#2	8	1,719,291	G/A	*CLN8*	R24H	5.07	Father

WES, whole-exome sequencing; ASD, autism spectrum disorder; GERP, Genomic Evolutionary Rate Profiling.

^a^Position according to GRCh37.

^b^Reference/mutant allele.

Next, we performed a two-stage follow-up study of the six novel rare missense variations identified by WES (Table 4). The genotypes determined by the TaqMan assay were identical to those determined using Sanger sequencing in the probands from families #1 and #2. In the Niigata sample, heterozygous *CLN8* R24H was identified in a male patient with autistic disorder and selective mutism and in a male control. In these individuals, *CLN8* R24H was confirmed by Sanger sequencing. The mutant H allele frequency was higher in patients (0.0021) than in controls (0.0007), although the association was not significant. In the heterozygous patient, the mutant H allele was not transmitted from his unaffected mother. Genomic DNA samples from his father, of unknown affection status, were not available; therefore, we were unable to confirm whether the father is heterozygous for *CLN8* R24H. *C1S* P19 and *TRAPPC12* E297Q were detected in controls, but not in patients. The other three variations (*SLC7A11* G77S, *ICA1* G167A, and *DNAJC1* A508G) were not identified in patients or controls. *CLN8* R24H was further genotyped in the Nagoya sample, but was not detected in patients or controls. When we combined the Niigata and Nagoya samples, there was no significant association between *CLN8* R24H and ASD.

**Table 4:** Genotyping of the six novel rare missense variations in the follow-up study.

Sample	Gene	Protein	ASD				Control				Allelic *p*	OR	95% CI
			1/1^a^	1/2^a^	2/2^a^	MAF	1/1^a^	1/2^a^	2/2^a^	MAF			
Niigata	*SLC7A11*	G77S	241	0	0	0	667	0	0	0	-	-	-
	*ICA1*	G167A	240	0	0	0	667	0	0	0	-	-	-
	*DNAJC1*	A508G	240	0	0	0	667	0	0	0	-	-	-
	*C1S*	P197S	240	0	0	0	665	2	0	0.0015	1.00	0	-
	*TRAPPC12*	E297Q	240	0	0	0	666	1	0	0.0007	1.00	0	-
	*CLN8*	R24H	240	1	0	0.0021	666	1	0	0.0007	0.46	2.8	0.2–44.4
Nagoya	*CLN8*	R24H	309	0	0	0	350	0	0	0	-	-	-
Combined	*CLN8*	R24H	549	1	0	0.0009	1016	1	0	0.0005	1.00	1.8	0.1–29.6

ASD, autism spectrum disorder; CI, confidence interval; MAF, mutant allele frequency; OR, odds ratio.

^a^Genotypes: reference and mutant alleles are denoted by 1 and 2, respectively.

*CLN8* R24H was identified in five affected and one unaffected individuals (Table 5). All were male. We observed social impairments in all five affected individuals. The father from Family #2 had difficulties in maintaining social relationships. Sibling #1 had difficulties in collaborating with peers and in maintaining friendships. Siblings #2 and #3 exhibited poor eye contact, problems with sharing interests, and difficulties in maintaining friendships. Patient #1 from the Niigata sample showed one-sided social interactions and a lack of speech. Restricted repetitive behaviors and interests were observed in all affected individuals except for the father. Patient #1 also had comorbid selective mutism. Intelligence quotient (IQ) data were available for three ASD siblings from family #2 and indicated borderline intellectual functioning in sibling #3 (full-scale IQ = 73). The father, sibling #2 and patient #1 had epilepsy.

**Table 5:** Clinical phenotypes of the six individuals heterozygous for *CLN8* R24H.

Clinical phenotype	Family #2				Niigata	
	Father	Sibling #1	Sibling #2	Sibling #3	Patient #1	Control #1
Transmission	Unknown	Paternal	Paternal	Paternal	Paternal or *de novo*	Unknown
Sex	Male	Male	Male	Male	Male	Male
Age	43	13	11	8	9	35
DSM-IV Diagnosis	PDD-NOS	Asperger	Asperger	Asperger	Autism	-
Social impairments	+	+	+	+	+	-
Communication impairments	-	-	-	-	+	-
Restricted repetitive behaviors and interests	-	+	+	+	+	-
Comorbidity	-	-	-	-	Selective mutism	-
Full-scale IQ	No data	96	80	73	No data	No data
Epilepsy	+	-	+	-	+	-

IQ, intelligence quotient; PDD-NOS, pervasive developmental disorder not otherwise specified.

## Discussion

In the present study, we identified six novel rare missense variations by WES in two ASD families, each with three affected siblings. Among these variations, only *CLN8* R24H co-segregated with ASD in family #2. Also, in the follow-up study, *CLN8* R24H was the only variation to have a higher mutant allele frequency in patients (0.09%) compared with controls (0.05%), although the association was not significant. Among six male *CLN8* R24H heterozygotes, five were affected with ASD. Of note, a terminal deletion at 8p23.2-pter, including *CLN8*, was identified in a Han Chinese boy with autism, epilepsy and severe intellectual disability [8]. Taken together, these findings suggest that *CLN8* is a potential genetic risk factor for ASD.

Certain homozygous or compound heterozygous *CLN8* mutations cause two distinct variants of neuronal ceroid lipofuscinosis-8 (CLN8; OMIM#600143): Northern epilepsy variant (OMIM#610003), also known as progressive epilepsy with mental retardation (EPMR), and a more severe form of variant late-infantile neuronal ceroid lipofuscinosis (vLINC). For example, homozygous R24G was identified in 22 Finnish patients with EPMR [9], while homozygous or compound heterozygous mutations (L16M, A30fs20X, T170M, R204C, and W263C) were found in Turkish patients with vLINC [10]. In the present study, we identified the heterozygous R24G variant in six Japanese individuals. They had no personal or family history of CLN8. Several other heterozygous *CLN8* missense variations have been identified by WES in 1208 Japanese individuals. These have been registered at http://www.genome.med.kyoto-u.ac.jp/SnpDB/. However, these variations and Japanese patients are not registered in the neuronal ceroid lipofuscinosis (NCL) Mutation and Patient Database (http://www.ucl.ac.uk/ncl/mutation.shtml).

*CLN8* encodes CLN8, which has five transmembrane domains, a TRAM-LAG1-CLN8 (TCL) domain, and a C-terminal endoplasmic reticulum (ER)-retrieval signal [11]. In non-neuronal cells, CLN8 is an ER resident protein that recycles between the ER and the ER-Golgi intermediate compartment (ERGIC) using the C-terminal ER-retrieval signal [12]. In neuronal cells, CLN8 is also localized to the ER and ERGIC [13, 14]. CLN8 plays a role in cell proliferation during neuronal differentiation and in protection against neuronal cell apoptosis [14]. Taking these findings into account, we hypothesize that *CLN8* R24H may cause abnormal neural cell proliferation and/or death, which results in ASD development [15]. Furthermore, the functional effect of R24H is predicted to be ‘probably damaging’ by PolyPhen-2 (http://genetics.bwh.harvard.edu/pph2/), but ‘tolerated’ by SIFT (http://sift.bii.a-star.edu.sg/index.html). Nevertheless, the functional implications of *CLN8* R24H in ASD remain to be elucidated.

We recognize the limitations of this study. First, our sample size in the follow-up study may not provide adequate statistical power to detect an association between *CLN8* R24H and ASD, because the risk allele frequency was extremely low. In the combined sample comprising the Niigata and Nagoya samples, the H allele frequencies were 1/1100 in patients and 1/2034 in controls, and the association was not significant (odds ratio = 1.8, 95% confidence interval = 0.1–29.6). If the genotypic relative risk is set to 1.8 for heterozygous risk allele carriers under the dominant model of inheritance, approximately 34,000 patients and 34,000 controls are needed to adequately detect association with a power of 0.80. Second, the patient and control groups in the Niigata and Nagoya samples were not age- or sex-matched. Prevalence of ASD is higher in males than in females [16], and *CLN8* R24H was identified only in males. Therefore, we analyzed males but did not find a significant association (data not shown). Third, there were differences in the percentages of the three diagnostic subgroups (autistic disorder, Asperger’s disorder, and PDD-NOS) between the Niigata and Nagoya samples. These differences may affect the mutant allele frequency of *CLN8* R24H in the samples, although this variation was identified in all three diagnostic subgroups. Fourth, our participants were not assessed using standardized structured interviews. However, a diagnosis of ASD was assigned on the basis of all available sources of information, including unstructured interviews of patients and their families, clinical observations, and medical records. Controls showed good social and occupational skills, but were not well characterized. Fein *et al*. [17] have described individuals with a history of ASD but who no longer show any significant autistic impairments. Therefore, we cannot exclude the possibility that our controls may include some individuals with early histories of ASD.

In conclusion, our present study suggests that *CLN8* R24H plays a role in the genetic etiology of ASD, at least in a subset of ASD patients.

## Supporting Information

S1 Table Demographic data of the Niigata and Nagoya samples. (DOC)

S2 Table Sequences of primers used for Sanger sequencing. (DOC)

S3 Table Probes used for TaqMan SNP assays. (DOC)
