# Supplementary material for: Novel Rare Missense Variations and Risk of Autism Spectrum Disorder: Whole-Exome Sequencing in Two Families with Affected Siblings and a Two-Stage Follow-Up Study in a Japanese Population
Source: PLoS One. 2015 Mar 25;10(3):e0119413. doi: 10.1371/journal.pone.0119413 (PMC4373693; doi:10.1371/journal.pone.0119413)
Supplement: S1 Table — (DOC) [file pone.0119413.s001.doc]

**Table S1. Demographic data of the follow-up study**

| Demographic data | Patient | Control |
| --- | --- | --- |
| Niigata sample |  |  |
| Male/female (%) | 189/52 (78.4/21.6) | 341/326 (51.1/48.9) |
| Mean age ± SD (years) | 18.1 ± 8.5 | 38.3 ± 10.8 |
| Autism/Asperger/PDD-NOS (%) | 72/105/64 (29.9/43.6/26.5) | - |
| Nagoya sample |  |  |
| Male/female (%) | 236/76 (75.6/24.4) | 109/243 (31.0/69.0) |
| Mean age ± SD (years) | 19.6 ± 10.2 | 45.9 ± 10.8 |
| Autism/Asperger/PDD-NOS (%) | 131/89/92 (42.0/28.5/29.5) | - |

SD, standard deviation; PDD-NOS, pervasive developmental disorder not otherwise specified
