# Supplementary material for: Novel Rare Missense Variations and Risk of Autism Spectrum Disorder: Whole-Exome Sequencing in Two Families with Affected Siblings and a Two-Stage Follow-Up Study in a Japanese Population
Source: PLoS One. 2015 Mar 25;10(3):e0119413. doi: 10.1371/journal.pone.0119413 (PMC4373693; doi:10.1371/journal.pone.0119413)
Supplement: S2 Table — (DOC) [file pone.0119413.s002.doc]

**Table S2. Sequences of primers used for Sanger sequencing**

| Gene | Protein | Forward | Reverse |
| --- | --- | --- | --- |
| *SLC7A11* | G77S | 5'-CTGCAGGGAAATGTTAACGGG-3' | 5'-ATGCTGCATCTGGGTAGTTCA-3' |
| *ICA1* | G167A | 5'-CTTTTCGGCATCGGGCCAT-3' | 5'-TTACCTTCCAGCAGGTACCCC-3' |
| *DNAJC1* | A508G | 5'-GCAGAACAAAACGAGTCCAGC-3' | 5'-TGCACAGATTCACACGCTCTA-3' |
| *C1S* | P197S | 5'-TCTATCCCTCCAGTTAGGGCAG-3' | 5'-TCAAGGCAGTTTCCCGCTG-3' |
| *TRAPPC12* | E297Q | 5'-AGCCTGAATGCAAAACGCAG-3' | 5'-TGGACTGGAGAGAGAGAGCG-3' |
| *CLN8* | R24H | 5'-ACAATGAATCCTGCGAGCGA-3' | 5'-AAGAATCCCGTTGCTGTCGT-3' |
