# Supplementary material for: Novel Rare Missense Variations and Risk of Autism Spectrum Disorder: Whole-Exome Sequencing in Two Families with Affected Siblings and a Two-Stage Follow-Up Study in a Japanese Population
Source: PLoS One. 2015 Mar 25;10(3):e0119413. doi: 10.1371/journal.pone.0119413 (PMC4373693; doi:10.1371/journal.pone.0119413)
Supplement: S3 Table — (DOC) [file pone.0119413.s003.doc]

**Table S3. Probes used for TaqMan SNP assays**

| Gene | Protein | Forward primer | Reverse primer | Reporter 1 | Reporter 2 |
| --- | --- | --- | --- | --- | --- |
| *SLC7A11* | G77S | 5'-GGGCGTGCTCCAGAACA-3' | 5'-ACACCGTCCAGATGGTCAGA-3' | 5'-VIC-CAGCGTGGGCATGT-NFQ-3' | 5'-FAM-CAGCGTGAGCATGT-NFQ-3' |
| *ICA1* | G167A | 5'-GGTGAACCGCATGGAACAGT-3' | 5'-CTGAGACACGTCCTTCATCCATAAT-3' | 5'-VIC-CAGGACGGAATATAGAGGAG-NFQ-3' | 5'-FAM-AGGACGGAATATAGAGCAG-NFQ-3' |
| *DNAJC1* | A508G | 5'-CGTGGACTCAAAATCAACAGAAACT-3' | 5'-AGAGGATCCCCTTGGGTACTG-3' | 5'-VIC-CTGCAACGCCAGTTCNFQ-3' | 5'-FAM-CTGCAACCCCAGTTC-NFQ-3' |
| *C1S* | P197S | 5'-GATTCTCCCTTTCTCTTTTTCTCTGTTAGT-3' | 5'-CCAACCGGATCTGGTATTCACA-3' | 5'-VIC-TTGAGTTCTCTGGATATGGT-NFQ-3' | 5'-FAM-TTGAGTTCTCTGAATATGGT-NFQ-3' |
| *TRAPPC12* | E297Q | 5'-TCGGGCAGAATAACTTTGCAGAA-3' | 5'-GATGGAGGATCCTGCAGCTT-3' | 5'-VIC-TACCACTGCGTTTCTT-NFQ-3' | 5'-FAM-ACCACTGTGTTTCTT-NFQ-3' |
| *CLN8* | R24H | 5'-ATTTTTGACCTGGACTATGCATCCT-3' | 5'-CAAAGCCAGCGACCATCAG-3' | 5'-VIC-CGTGGAGCGGATCC-NFQ-3' | 5'-FAM-CGTGGAGTGGATCC-NFQ-3' |
